# Cost-Effective Design of Polarization and Bandwidth Reconfigurable Millimeter-Wave Loop Antenna

**DOI:** 10.3390/s23249628

**Published:** 2023-12-05

**Authors:** Rawad Asfour, Salam K. Khamas, Edward A. Ball

**Affiliations:** Communications Research Group, Department of Electronic and Electrical Engineering, University of Sheffield, Mappin Steet, Sheffield S1 3JD, UK; rwsasfour1@sheffield.ac.uk (R.A.); e.a.ball@sheffield.ac.uk (E.A.B.)

**Keywords:** circular polarization, loop antenna, millimeter wave, reconfigurable antenna

## Abstract

A singly fed reconfigurable circular loop antenna is proposed for millimeter-wave (mmWave) communication systems. This antenna’s distinctive feature lies in its capacity to adjust both polarization and bandwidth characteristics, a capability made possible by the strategic integration of two PIN diodes. These diodes are engineered to function in various modes, allowing for three distinct polarization states and accommodating two distinct bandwidths. A meticulous alignment of these PIN diodes enables the utilization of a single DC bias network as a highly effective RF choke, which simplifies the design and reduces the associated losses. Additionally, a planar biasing network that consists of coplanar strip-lines (CPS) has been employed eliminating the need for lumped elements. The simple and totally planar configuration offers a choice of right-hand circularly polarized (RHCP) radiation or left-hand circularly polarized (LHCP) radiation at 28 GHz. This is accompanied by impedance matching and axial ratio (AR) bandwidths of 12.9% and 8%, respectively, over the same frequency range with a gain of 7.5 dBic. Moreover, when the PIN diodes are unbiased, the antenna offers linear polarization (LP) over two narrow bandwidths at 27 GHz and 29 GHz featuring a maximum gain of 7.2 dBic. Therefore, the proposed configuration offers three operating modes: wide-band RHCP, wide-band-LHCP, and LP over dual narrow bands. Significantly, simulated results closely align with the measured outcomes, affirming the robustness and accuracy of this design.

## 1. Introduction

In the realm of mmWave applications, the demand for enhanced communication capabilities has fueled the emergence of reconfigurable antennas as a pivotal solution. These antennas offer a crucial degree of flexibility and adaptability, specifically tailored to address the challenges inherent in mmWave frequencies. Given that mmWave frequencies pose unique hurdles in signal propagation and susceptibility to environmental obstacles, reconfigurable antennas play a vital role in mitigating these challenges [1,2,3]. Their ability to adjust radiation patterns, polarization, frequency, and bandwidth characteristics in real-time provides a dynamic response to the intricacies of mmWave communication environments.

Moreover, beyond their technical advantages, reconfigurable antennas significantly contribute to the overall efficiency and cost-effectiveness of mmWave systems. By offering adaptability and versatility, these antennas facilitate streamlined design approaches, diminishing the reliance on complex and specialized hardware components. This, in turn, promotes the development of compact and lightweight mmWave devices, aligning with the escalating demand for miniaturization in contemporary communication systems.

In a parallel development, circularly polarized antennas have garnered substantial interest in the realm of mmWave communication systems. This interest is rooted in well-established advantages, such as their resistance to multipath interference and tolerance to misalignment between transmitting and receiving antennas [4]. As such, the incorporation of circularly polarized antennas into mmWave designs represents a strategic choice to enhance the robustness and reliability of communication systems operating in these high-frequency bands. The synergy between reconfigurable antennas and circularly polarized antennas presents a holistic approach to addressing the multifaceted challenges and optimizing performance in the rapidly evolving landscape of mmWave applications. However, circular polarization can be achieved in one of two senses: LHCP or RHCP. As a result, it is important to design an antenna that supports the two polarization senses to sustain communications in challenging and demanding environments. Therefore, studies have been published proposing novel mmWave circularly polarized antennas [5,6,7,8]. However, these approaches share a limitation of providing fixed circular polarization senses, i.e., either LHCP or RHCP, thereby restricting their usage to a single polarization type. In contrast, an array that utilizes LHCP and RHCP has been proposed [9]. Besides, other designs have been reported that incorporate multiple feeding ports to generate the desired circular polarization sense, albeit at the cost of added complexity [10,11]. In addition, a cost-effective design of a polarization reconfigurable mmWave antenna that is capable of radiating CP and LP waves provides another needed degree of flexibility to any communication system.

Prototypes of polarization reconfigurable mmWave antennas have been reported in several studies [12,13,14,15,16,17,18]. For example, a K-band polarization reconfigurable patch antenna has been proposed in a layered structure using RF-MEMS to switch between two polarization modes, LHCP and LP, over impedance bandwidths of 11.8% and 3%, respectively, with a peak gain of ~3.9 dBic [12]. Another polarization reconfigurable patch antenna that operates at 29 GHz has been proposed with LP, LHCP, and RHCP radiation modes [13]. The findings demonstrated respective impedance bandwidths of 5.1% and 3.1% for the CP and LP modes in combination with an AR bandwidth of 1.7% and a maximum gain of ~8.5 dBi in the LP mode. However, the reconfigurability relies on external stimuli, using UV laser pulses, to control the phase change of the Germanium Telluride (GeTe) material, which limits the practicality and poses manufacturing challenges, thereby detracting from its potential implementation. In a more recent study, 4 PIN diodes were utilized in the design of a reconfigurable patch antenna with three polarization modes RCHP, LHCP, and LP operating at 29 GHz with an impedance bandwidth of ~5.4% in all cases [14]. The measured respective gains are 3 dBic and 4 dBi in the CP and LP radiations albeit with no data for the AR bandwidths.

On the other hand, a 2 × 2 mmWave polarization reconfigurable patch antenna array has been reported by utilizing two PIN-diode pairs to switch between dual CP modes with respective impedance and AR bandwidths of 11% and 4% with an efficiency of 51% [15]. Besides, linear, and square arrays with 10 and 2 × 2 T-shaped slot elements have been reported with respective impedance bandwidths of 3.3% and 10%, where two mechanically switchable CP senses have been achieved over an AR bandwidth of 3% for both arrays [16]. Similarly, full polarization reconfigurability has been achieved using a phased array of 8 × 12 Butterfly elements with 4 feeding ports that switch the polarization by utilizing the required excitation amplitude and phase [17]. The AR and impedance are presented over a frequency range of 27–29 GHz with a simulated total efficiency of 56–60%. Furthermore, a double-folded, polarization-reconfigurable, dual-antenna array has been proposed with a CP switching over a bandwidth of 7% through the activation of a single-pole-double-throw (SPDT) switch when 10 elements are used [18]. In addition, a figure of merit was introduced and used to compare the performance to those in earlier studies. However, in [15,16,17,18], polarization reconfigurability has been achieved by utilizing a substantial number of elements in conjunction with customized feeding networks that naturally increase the cost and complexity compared to a single antenna configuration.

In the presented study three modes of polarization reconfigurability are achieved using a singly fed open-loop antenna that incorporates two PIN diodes only. In addition, the antenna offers impedance and CP bandwidths of 12.9% and 7%, respectively, in combination with a gain of ~8.5 dBic and an estimated total efficiency of 79%. Moreover, the proposed configuration avoids the need to utilize UV laser pulses, multi-layer PCB structures, or large arrays with complex feeding networks. 

It should be noted that the RHCP and LHCP modes have been achieved when a single PIN diode is forward-biased while the other PIN diode is reverse-biased. On the other hand, when both PIN diodes are under zero-bias conditions, the LP mode is achieved with dual narrow impedance bandwidths since the total impedance presented by the two unbiased diodes at the input of the antenna is different from that presented using one forward, and one reverse, biased PIN diodes. Therefore, the proposed configuration offers the distinct advantage of offering hybrid reconfigurability by varying polarization and bandwidth without any compromise on the performance. To the best of the authors’ knowledge, this is the first attempt to design a mmWave antenna that offers hybrid reconfiguration. It may be worth pointing out that bandwidth reconfigurability received increased interest in recent years since an antenna with bandwidth control could partially perform a filtering task as well as the radiation [19,20,21]. 

The effectiveness of the proposed antenna design is demonstrated through comprehensive simulation and measurement results. These results showcase promising performance in terms of impedance and axial ratio bandwidths. The outcome of this study highlights the potential usefulness of the proposed CP reconfigurable antenna for mmWave applications, as well as practical implementations in satellite and wireless communications systems.

The paper is organized as follows: Section 2 summarizes the key contributions and Section 3 introduces the utilized configuration and design principles of the polarization reconfigurable loop antenna. Section 4 describes the utilized PIN diodes, and DC biasing network while Section 5 presents the switching mechanism of the polarization modes. It should be noted that a single biasing network has been utilized for the two PIN diodes, which provides further simplicity in the design. Moving on to Section 6, details are provided concerning the prototypes, fabrication processes, and measurements of the proposed antenna. Subsequently, Section 7 provides a comparison between the performance of the proposed antenna against those reported in the literature. Section 8 presents the conclusion comments.

## 2. Contribution

Considering the challenges mentioned above, the key contributions of the presented work include the design and fabrication of a cost-effective mmWave polarization reconfigurable planar loop antenna. This is combined with a reconfigurable bandwidth, which means the proposed design offers hybrid reconfigurability using only two PIN diodes. As a result, the antenna offers three operating modes: LHCP and RHCP radiations over a wide bandwidth, and LP radiation over narrow dual bands. This capability enables the antenna to produce various types of polarization, enhancing its versatility and adaptability for different communication needs. Besides, a coplanar strip line (CPS) structure has been employed featuring a photonic bandgap (PBG) section to facilitate the biasing of the PIN diodes with no need for lumped RF chokes or capacitors, which eliminates additional losses introduced by these elements, and hence results in higher efficiency.

## 3. Antenna Configuration

**Figure 1:**
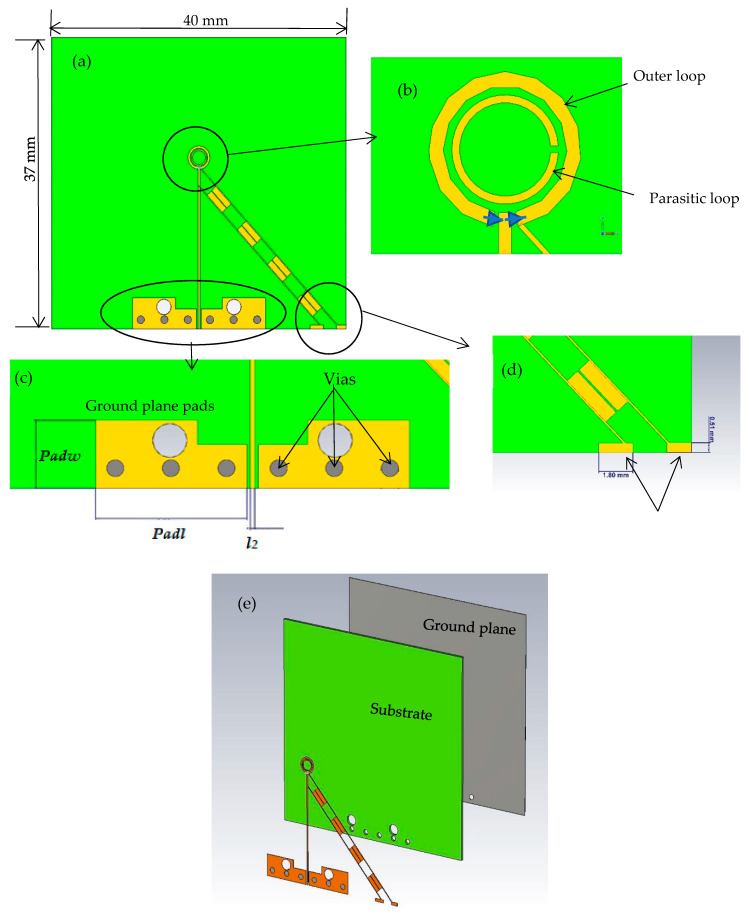
The proposed reconfigurable loop antenna with two PIN diodes and the corresponding biasing network (**a**) 2D view. (**b**) outer and parasitic loops. (**c**) ground plane pads. (**d**) pads for soldering (**e**) 3D view.

**Figure 2:**
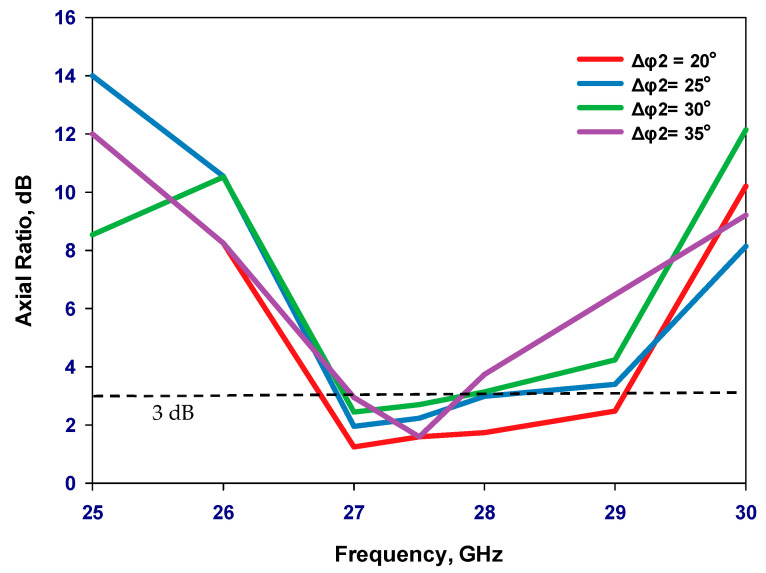
Axial ratio variation with different sizes of outer loop’s gap.

**Figure 3:**
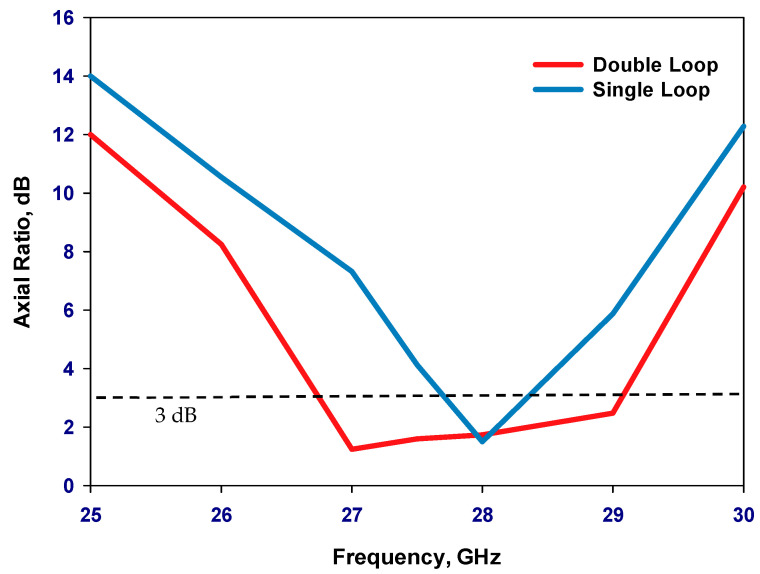
Simulated axial ratio and gain of single and double loop antennas.

Figure 1 presents the configurations of a reconfigurable antenna designed to operate at 28 GHz. The proposed antenna consists of two concentric loops [22,23,24] in which the outer and inner loops represent the active and parasitic elements, respectively. The proposed antenna is printed on the upper surface of the Rogers RO4003C dielectric substrate, with an equal-sized ground plane positioned beneath the substrate. Besides, the outer loop’s radius needs to be selected so that the circumference is approximately one effective wavelength, $\lambda_{eff}$. Two gaps have been created on the outer loop to house two PIN diodes that facilitate reconfigurability. The gaps have been optimized to create an optimal traveling wave current distribution along the loop, which is crucial for achieving circular polarization. Figure 2 illustrates the parametric analysis, showcasing the variation in the axial ratio for various values of Δ*φ*_2_ with the aim of identifying the most favorable angular gap that yields the widest axial ratio bandwidth. As a result, the optimum Δ*φ*_2_ has been determined as 20°. Besides, Figure 3 presents the simulated axial ratio, comparing two scenarios: with and without the parasitic loop. Upon utilizing the inner loop, the AR ≤ 3 dB bandwidth experiences a notable increase from 2.5% to 8.1%. As anticipated, the parasitic loop significantly enhances the AR bandwidth, given that each loop generates a single AR minimum point. The merging of these two minimum AR points results in the observed bandwidth improvement. The circumference of the outer loop can be calculated as

(1) $$\varepsilon_{eff}=\left(\varepsilon_{r}+1\right)/2.$$

(2) $$\lambda_{eff}=\lambda_{0}/\sqrt{\varepsilon_{eff}}$$

(3) $$2\pi R_{1}≃\;\lambda_{eff}$$

where $\varepsilon_{eff}$ is the effective relative permittivity, and *λ*_0_ is the free-space wavelength.

The two PIN diodes function as switches capable of toggling between forward-bias and reverse-bias states to select the desired polarization sense. For the utilized PIN diodes, the forward-bias resistance is set to 5.2 Ω, while the reverse-bias resistance is 15 kΩ. By adjusting the gaps Δ*φ*_1_ and Δ*φ*_2_ as well as controlling the PIN diode states, the polarization sense can be electronically switched between RHCP and LHCP over a relatively wide frequency range. In addition, LP radiation can be achieved when the two diodes are unbiased. The antenna is connected via a microstrip line at ϕ = 0, featuring a width designated by the parameter $l_{2}$. The microstrip line has been designed to attain a 50 Ω impedance and has been connected to the coaxial cable using the structure shown in Figure 1. In addition, two holes have been incorporated in the pads that are utilized for the SMA connector fixing. Furthermore, the integration of vias into the design yields several benefits, encompassing improved grounding, mitigation of surface waves, and the expansion of bandwidth, thereby enhancing overall antenna performance. Consequently, the presented antenna array is furnished with six vias, each possessing a radius of 1 mm and situated at 4 mm intervals, center to center. Detailed configuration parameters are summarized in Table 1.

## 4. DC Biasing Network

The PIN diode possesses distinctive electrical characteristics that render it an excellent choice for various switching applications. Its primary advantage lies in its capacity to alter its resistance based on the applied bias voltages, allowing it to seamlessly transition between conducting and non-conducting states. When incorporated in antennas, the PIN diode functions as a switch, facilitating the antenna’s ability to switch between different signal paths. In the proposed configuration, the MA4AGFCP910 PIN diode has been utilized [25]. It should be noted that the PIN diodes have been modeled in CST using lumped elements in the equivalent RLC circuits of the forward and reverse-biased diodes using the parameters presented in Table 2. 

**Figure 4:**
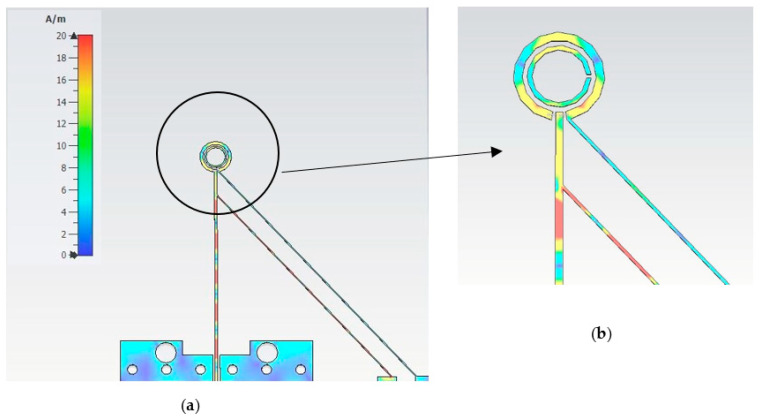
Surface current distribution of the proposed antenna with CPS bias line only at 28 GHz; (**a**) Whole configuration, (**b**) View of currents along the loop and adjacent line sections.

**Figure 5:**
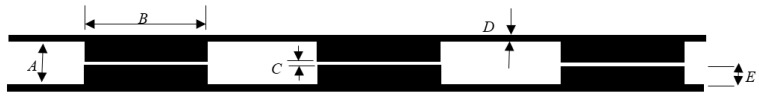
Structure of CPS bias incorporating the PBG.

As mentioned earlier, the PIN diodes have been placed in two strategically placed gaps incorporated within the outer loop. The PIN diodes are then subjected to a biasing process facilitated by a designated bias line. Figure 4 illustrates the proposed loop antenna that incorporates a single CPS to supply the DC bias current to the two PIN diodes. The surface current distribution of the antenna with a CPS bias line is presented in Figure 4b. A periodic current distribution is observed along the bias line due to the RF current leakage from the antenna. Such current leakage results in unwanted electromagnetic radiation and causes the bias line to function as a radiating element with fields that interfere with and distort those radiated by the antenna, particularly the CP radiation. This is in addition to altering the antenna’s key characteristics, including resonance frequency, input impedance, AR, gain, and radiation pattern. To mitigate this issue, the development of a novel bias line structure is imperative one that effectively carries the requisite DC bias current while preventing the unwanted flow of RF current. To curtail RF current propagation within the coplanar strip line, a PBG structure with a period of *λ_eff_*/4 has been integrated into the bias line, as illustrated in Figure 5 [26], to present a high impedance that minimizes the leakage of the RF current along the CPS line. Therefore, the PBG structure effectively acts as an RF choke that suppresses the flow of RF current signal along the coplanar strip line, thereby ensuring optimal performance and efficiency. The PBG section consists of a sequence of high and low quarter wavelength impedance transformer sections. The gap between the lines of the high-impedance section is defined as *A*, the PBG cell’s length is *B*, the gap between the thicker lines of the low-impedance PBG cells is *C*, and the widths of the CPS line are *D* and *E*. The design parameters for the CPS bias line and PBG are summarized in Table 3. 

Employing the CPS-PBG facilitates the biasing of the PIN diodes with no need for lumped chokes or capacitors, which eliminates additional losses introduced by these components, and hence results in higher efficiency. Furthermore, to achieve a design that optimizes cost-effectiveness and efficiency while eliminating the need for an additional feeding network to bias the second PIN diode, a modification was introduced by aligning both diodes in the same direction. Such an arrangement ensures that a single switch and a single biasing circuit are sufficient to achieve reconfigurability, which simplifies the design, improves efficiency, and reduces the required DC power. It should be noted that the biasing CPS-PBG line has been tilted by 45° to avoid sharp corners that exist if vertical and horizontal sections are utilized for biasing.

**Figure 6:**
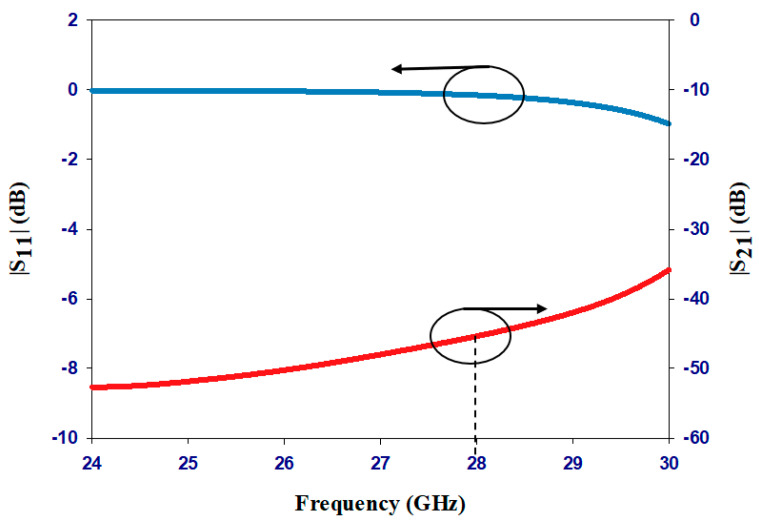
The reflection and transmission coefficients of the RF choke in the proposed design.

**Figure 7:**
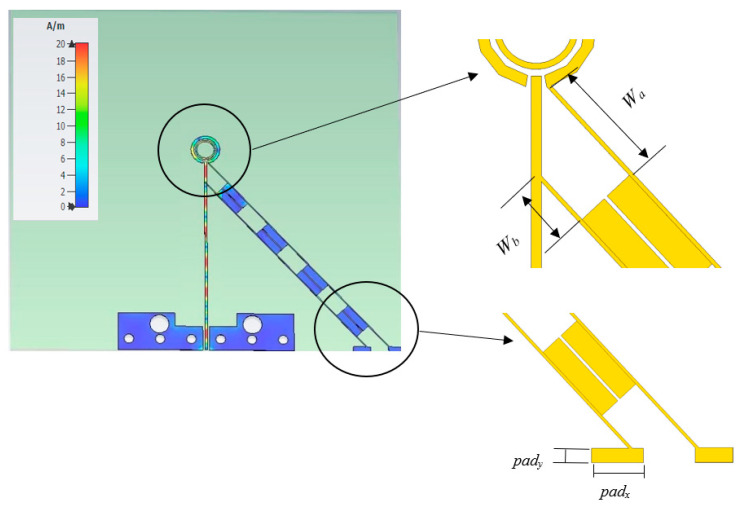
Surface current distribution of the proposed antenna with CPS bias line and PBG structure at 28 GHz.

Figure 6 showcases the reflection and transmission coefficients of the RF choke through the utilization of two ports positioned at each end of the biasing line. Notably, the stopband of the proposed RF choke encompasses the antenna’s operating bandwidth. On the other hand, Figure 7 presents the antenna’s surface current distribution along the CPS-PBG bias line at 28 GHz, where it can be noted that adding the PBG sections effectively cuts off the RF current from flowing across the CPS line, which means the CPS-PBG line serves the purpose as a distributed RF choke. Furthermore, as can be observed from Figure 7, the biasing network has been connected to the circular loop using strip line sections with lengths of *W_a_* and *W_b_* that have been adjusted for optimum matching. 

**Figure 8:**
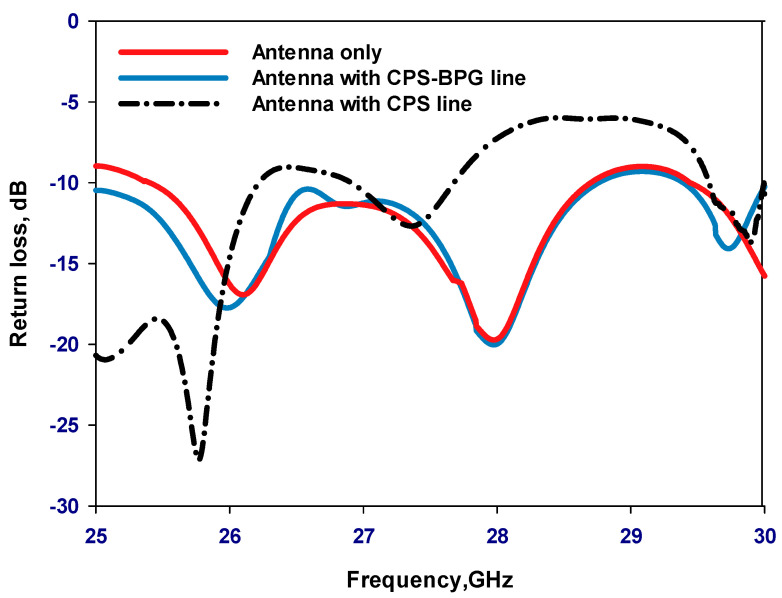
Reflection coefficients for various antenna configurations.

**Figure 9:**
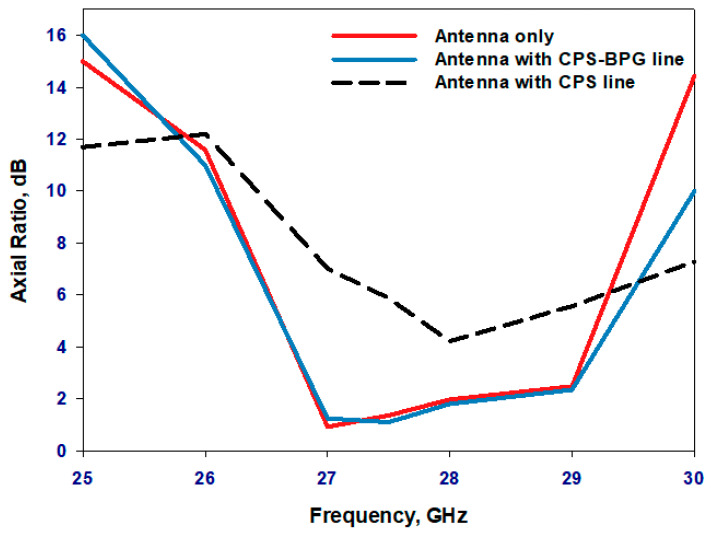
The axial ratio for various antenna configurations.

Figure 8 and Figure 9 present the variations of reflection coefficient and axial ratio, respectively, for various antenna configurations; without any biasing, with the CPS line alone, and after the additions of the PBG to the CPS lines. It can be noted from these results that adding the CPS line without the PBG has deteriorated the performance significantly due to the undesired flow of the unbalanced RF current. On the other hand, the performance of a standing-alone antenna has been preserved by adding the PBG to the CPS line to achieve the equivalent of an RF choke that stops any RF current flowing along the biasing network over the desired frequency range. In addition, the antenna performance has been preserved across the operating bandwidth, which demonstrates that the CPS-PBG stopped the RF current leakage over a relatively wide frequency range compared to that of a traditional filter.

## 5. Polarization Switching by Utilizing Two PIN Diodes

**Figure 10:**
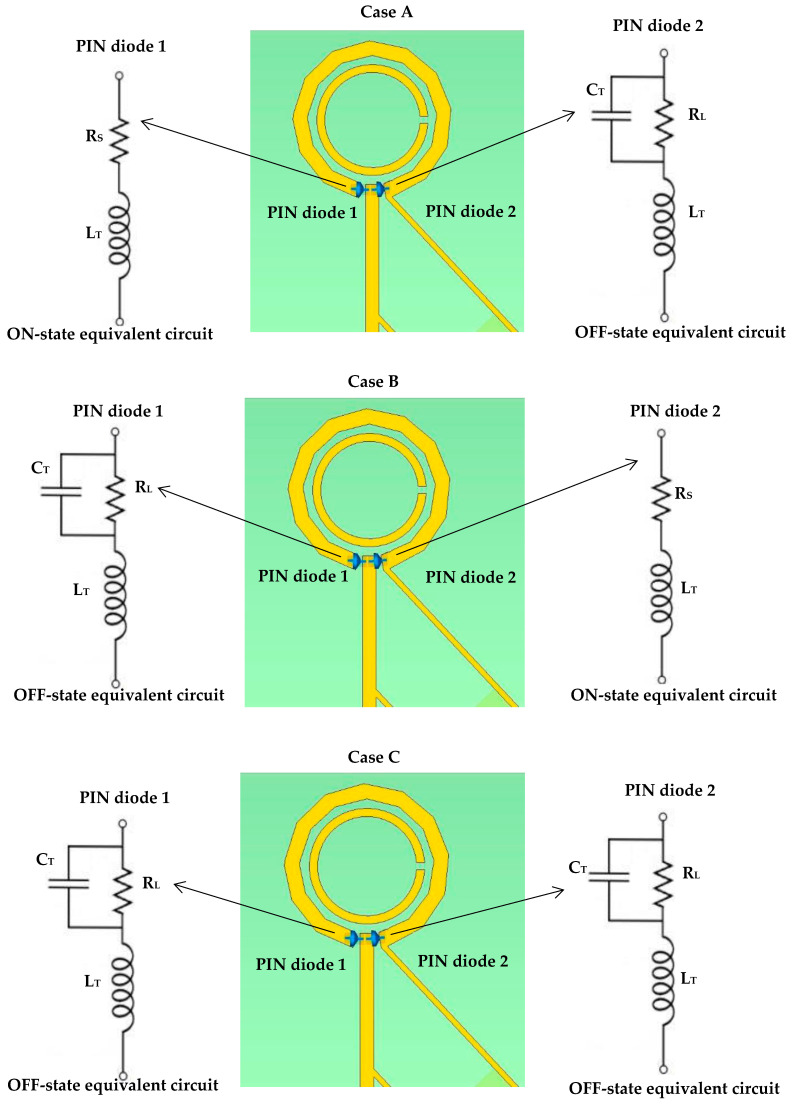
Structures of achieving different polarizations by using two PIN diodes. Case A: when the PIN diode 1 is forward biased, that is usually defined as the “ON” state. Case B: when PIN diode 2 is forward-biased. Case C: when the two PIN diodes are under zero-biased conditions.

Figure 10 presents the mechanism of switching the polarization state with the corresponding equivalent circuits, where three cases have been considered.

Case A: During this biasing, the resistance of PIN diode 1 decreases significantly, enabling easy flow of current across the gap. As a result, the current passes through the outer loop and reaches the reverse biased PIN diode 2, which is in the “OFF” state in which the diode resistance increases considerably. This behavior resembles that of an open switch, preventing current from flowing through the PIN diode 2. In this case, LHCP is achieved since the current flows clockwise throughout the antenna.

Case B: Offers RHCP by switching the bias voltage in a manner where PIN diode 2 is forward-biased, while PIN diode 1 is reverse-biased, which changes the current’s direction to counterclockwise resulting in RHCP wave.

Case C: Offers LP radiation by keeping the two PIN diodes under zero-biased conditions.

It should be noted that the chosen alignment of the two PIN diodes simplifies the design and eliminates the need for an additional biasing network, thereby optimizing cost-effectiveness while achieving the desired polarization modes. In addition, with this alignment, it is not possible to have the two PIN diodes, neither forward, nor reverse, biased simultaneously. Hence the unbiased condition of the two PIN diodes has been utilized to achieve the LP polarization. Table 4 summarizes the states of the reconfigurable loop antenna, categorized according to various polarization states. 

**Figure 11:**
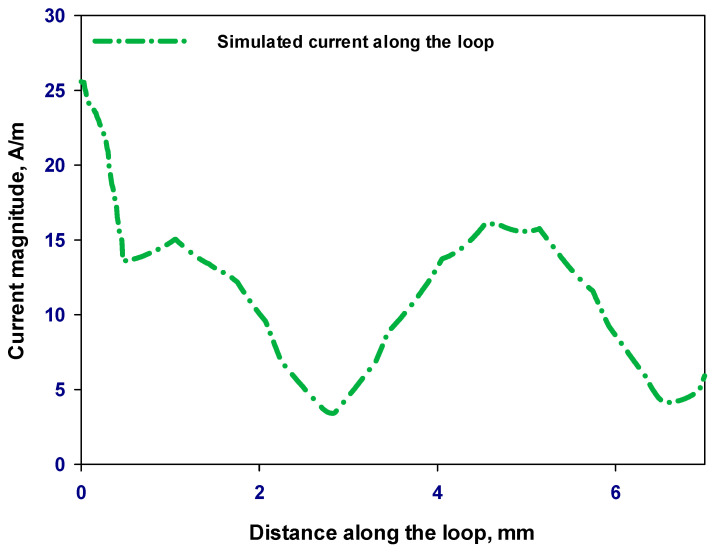
Travelling-wave current distribution along the loop’s circumference at 28 GHz.

Figure 11 illustrates the current distribution along the loop’s circumference at 28 GHz. In contrast to linearly polarized antennas, where the current’s amplitude fluctuates to form a standing wave along the length of the antenna, circularly polarized waves require a traveling wave current distribution to be radiated, where the current’s amplitude fluctuates along the loop, gradually decreasing as it reaches the other gap on the loop. 

## 6. Fabrication and Measurements

**Figure 12:**
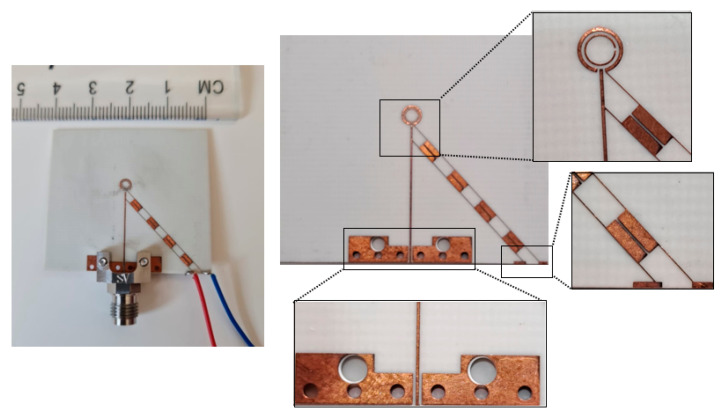
A prototype of the reconfigurable loop antenna.

**Figure 13:**
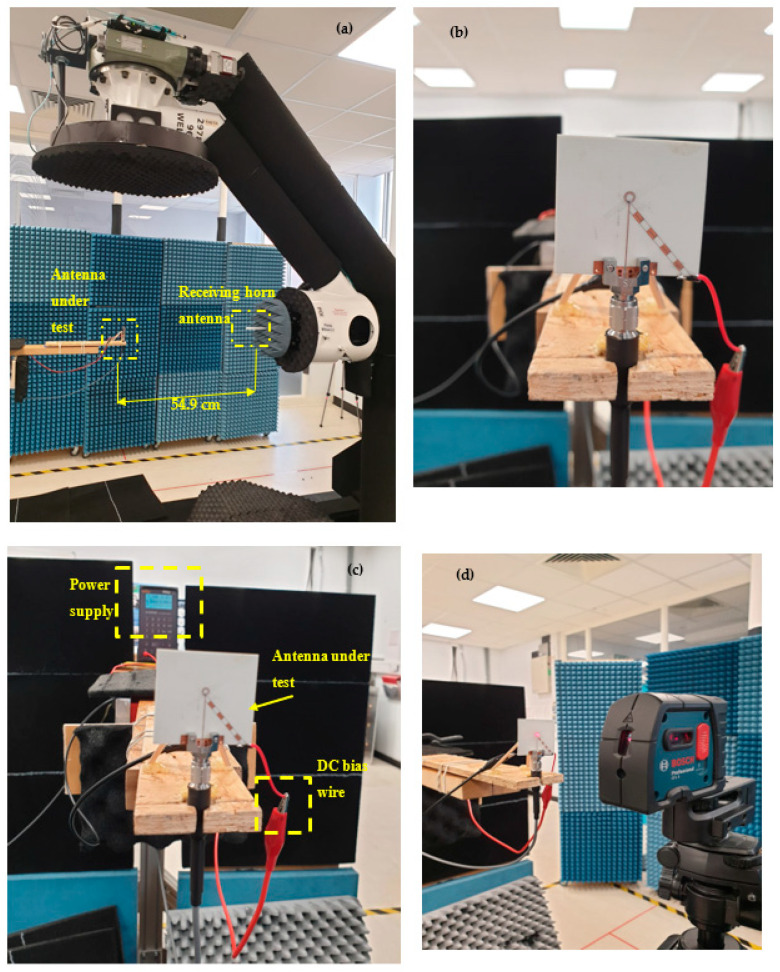
mmWave measurements setup; (**a**) Far field measurements, (**b**) top view, (**c**) Power supply calibration, (**d**) Antenna alignment.

A prototype of the antenna has been manufactured by Wrekin [27] as demonstrated in Figure 12, where a 2.4 mm SMA connector has been utilized. The overall antenna size is 40 mm × 37 mm. The vias were created by small holes in a printed circuit board (PCB) and then these holes were plated with gold to establish electrical connections between the top and bottom surfaces of the PCB. The measurements’ set-up is illustrated in Figure 13 with the mmWave reconfigurable loop antenna inside the testing environment. For the far-field measurements, the NSI-MI Technologies system was utilized, while the N5245B vector network analyzer (VNA) was used to quantify the return losses [28]. An 85052D calibration kit has been utilized for the measurements of the reflection coefficient. In addition, the antenna was positioned at 54.9 cm from the reference horn antenna to measure its radiation pattern. Before this measurement, the power supply was calibrated and linked to the antenna through wires to apply bias to the PIN diodes.

### 6.1. Circular Polarization States

**Figure 14:**
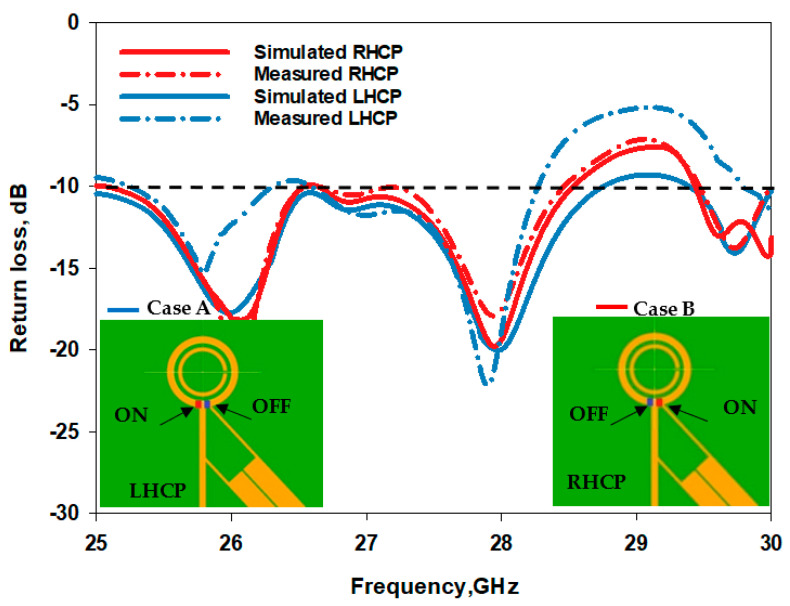
The simulated and measured return losses of the reconfigurable loop antenna.

**Figure 15:**
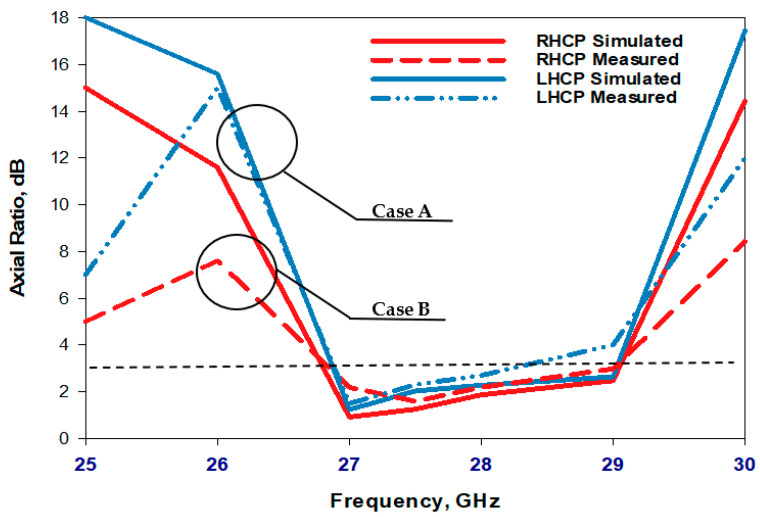
The axial ratio of the reconfigurable loop antenna for the two circular polarization senses.

**Figure 16:**
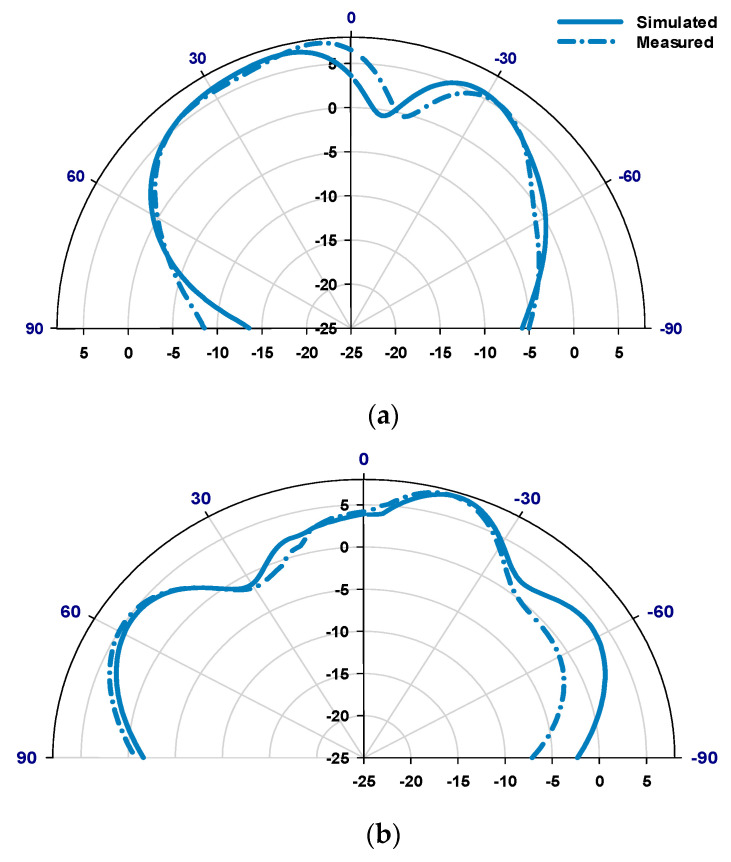
Realized gain patterns for the antenna in Case A at 28 GHz, (**a**) ϕ = 0°, (**b**) ϕ = 90°.

**Figure 17:**
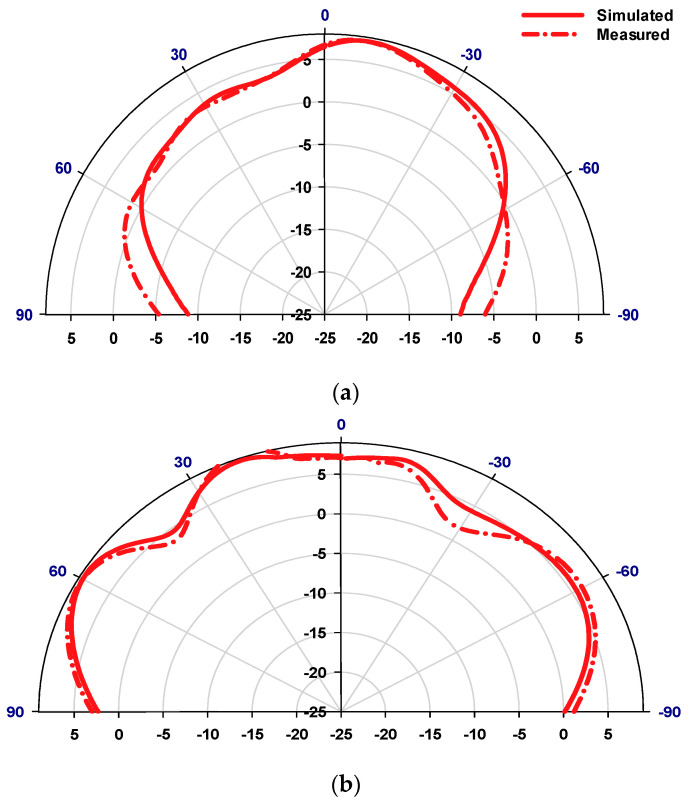
Radiation patterns for the antenna in Case B at 28 GHz, (**a**) ϕ = 0°, (**b**) ϕ = 90°.

Figure 14 illustrates a compelling comparison between the simulated and measured reflection coefficients, where the minimum S_11_ has been achieved at 28 GHz in all cases. This analysis delves deeply into the distinct cases of the proposed antenna with RHCP and LHCP modes. The simulation draws out intriguing details. Upon configuring the antenna in Case A, it achieves a 13.3% impedance bandwidth compared to 13.7% in Case B centered at 28 GHz in both cases. What adds credence to these findings is the alignment between measured and simulated impedance bandwidths. In Case A, the measurements mirror the simulation closely, showcasing a 12.7% impedance bandwidth, and in parallel, Case B achieves 11.2% bandwidth a strong testament to the reliability and consistency of the antenna’s performance across these divergent scenarios. The measured and simulated axial ratios are demonstrated in Figure 15 for both cases of LHCP and RHCP. In the scenario of Case A, the simulated 3 dB AR bandwidth covers approximately 8.1% within the frequency range of 26.75 to 29 GHz, effectively achieving LHCP radiation. The measured 3 dB AR bandwidth in Case A ranges from 26.85 to 28.9 GHz, with a bandwidth of circa 7.42% which is in close agreement with simulations. On the other hand, Case B offers a simulated 3 dB AR bandwidth of roughly 7.64%, spanning the frequency band from 26.89 to 29 GHz, while achieving the RHCP radiation. Concurrently, the corresponding measurement outcome registers around 60%, overlapping frequencies from 25 to 28.35 GHz. The simulated and measured realized gain radiation patterns for LHCP and RHCP senses are presented in Figure 16 and Figure 17 for cases A and B at 28 GHz. The measured radiation patterns mostly follow the simulated counterparts with slight differences due to the measurement errors at the mmWave frequency range. Remarkably, in both cases, the maximum radiation pattern is emitted in the broadside direction resulting in an LHCP wave in Case A and an RHCP wave in Case B. 

### 6.2. Linear Polarization State

**Figure 18:**
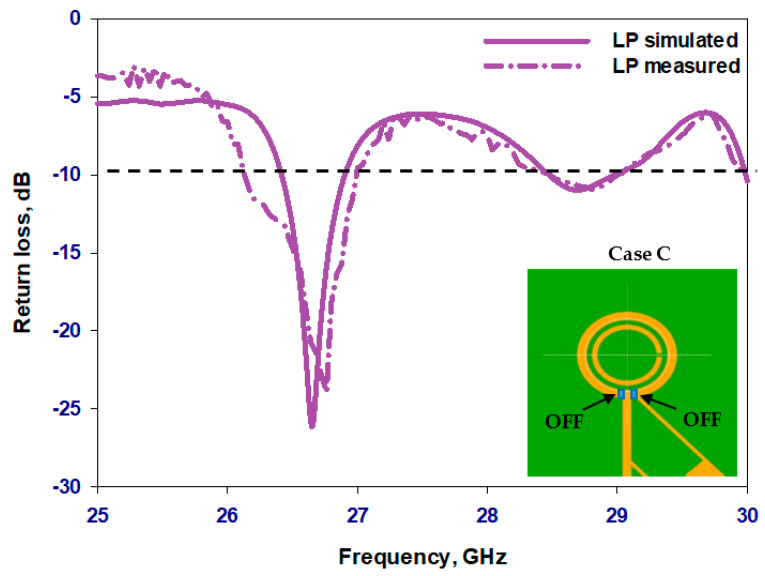
The simulated and measured return losses of the reconfigurable loop antenna operating in the LP state.

**Figure 19:**
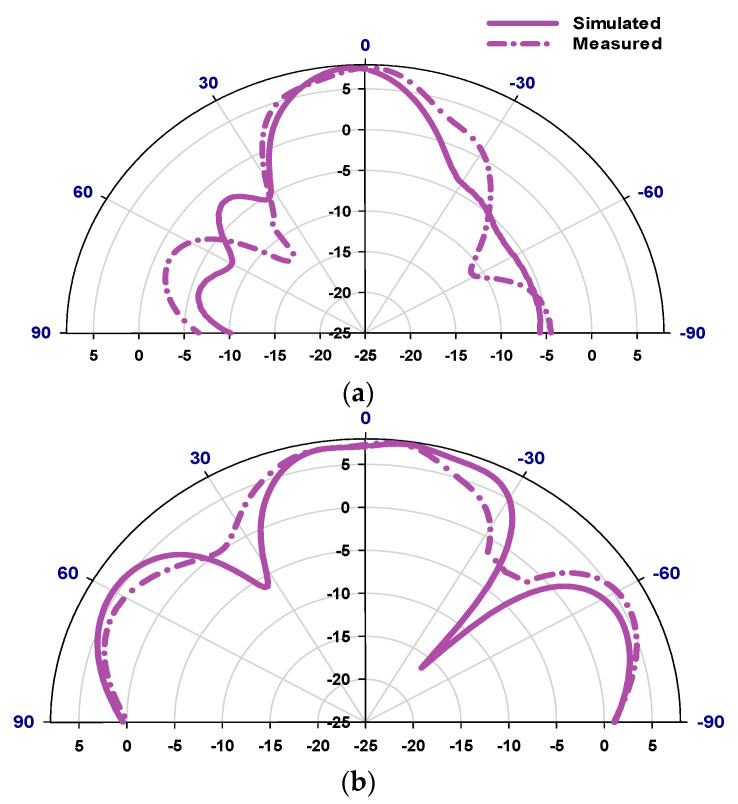
Radiation patterns for the reconfigurable antenna in Case C at 27 GHz; (**a**) ϕ = 0°, (**b**) ϕ = 90°.

Figure 18 presents the reflection coefficient when the two PIN diodes are under zero bias, where it can be noted that matching is achieved over dual narrow bands centered at 26.8 GHz and 29 GHz with considerably narrower bandwidths compared to that in the CP states. Namely, bandwidths of 3.4% and 2.1% have been achieved in the simulation compared to 2.3% and 1.9% in the measurements. The discrepancy can be attributed to the fact that the actual equivalent circuit of an unbiased PIN diode may be different from that of a reverse-biased PIN diode, which was used in the simulations. Figure 19 presents the simulated and measured realized gain patterns of the antenna within Case C at 27 GHz with close agreement. This configuration enables the LP radiation at different operating frequencies. 

**Figure 20:**
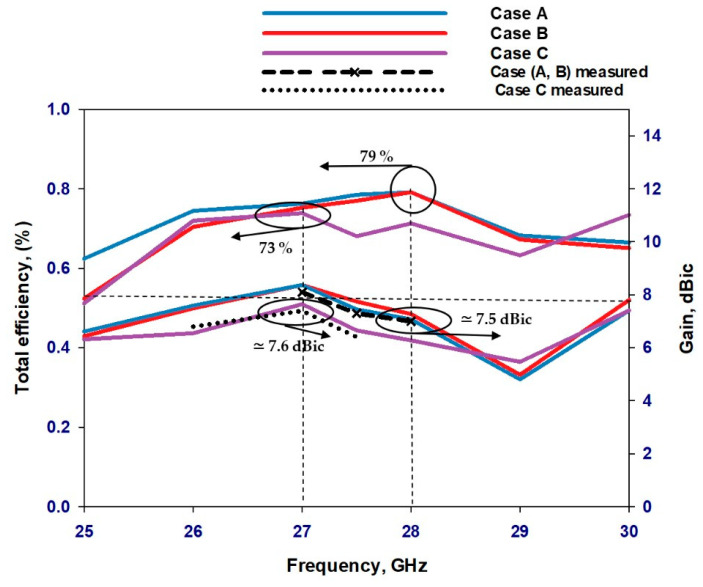
Total efficiency for polarization senses of the mmWave reconfigurable loop antenna.

Figure 20 illustrates the total efficiency and realized gain for the circular polarization and linear polarization modes. The simulated total efficiency remains consistent at circa 79% across the desired CP bandwidth, which demonstrates excellent performance for a reconfigurable antenna operating in the mmWave frequency range. The improvement in efficiency can be attributed to several factors such as the absence of lumped inductors and capacitors in the biasing network. This is in addition to utilizing a single biasing network for the two diodes instead of the common approach of utilizing a separate biasing network for each diode. This is in addition to employing a low-loss dielectric constant substrate. On the other hand, the PIN diode is associated with losses that have been accounted for in the achieved total efficiency of 79%. However, when considering Case C, it’s worth noting that the total efficiency at 27 GHz exhibits a slight reduction, to approximately 73%. This could be attributed to the higher resistances offered by the two PIN diodes. In addition, the comparison reveals a close correspondence between the measured and simulated realized gains. In Case A and Case B, the measured and simulated realized gain is circa 7.5 dBic at 28 GHz. On the other hand, Case C exhibits a realized gain of approximately 7.6 dB at 27 GHz.

## 7. Performance Comparison

Table 5 presents a comparison between the performance of the proposed antenna compared to polarization reconfigurable antenna prototypes that are reported in the literature. From the Table 5, it is evident that the presented antenna outperforms the single antenna prototypes reported in [12,13,14] in terms of the reported impedance and AR bandwidths as well as gain. In addition, the proposed antenna offers three polarization modes compared to two in [12], employs a simple switching mechanism compared to that utilized in [13], and needs only two PIN diodes compared to four in [14]. On the other hand, the studies in [15,16,17,18] are focused on polarization reconfigurable mmWave arrays, which naturally require a more complex design in terms of feed network and switching mechanisms for many antennas. However, the presented single-loop antenna offers wider impedance and CP bandwidths than those reported in [15,16,17] and a slightly narrower impedance bandwidth compared to [18]. In terms of the polarization modes, the arrays reported in [15,16] offer two polarization states compared to three in the present study. Although full polarization reconfigurability has been achieved in [17], four excitation ports were used, which increases the cost and complexity compared to the case of a singly fed antenna. The total efficiency of the presented design is higher than those achieved in [15,17,18] and close to that reported in [16]. On the other hand, higher gains have been reported in [16,17,18] due to the considerable number of utilized elements. The overall footprint of the proposed configuration has increased owing to the required length of the CPS-PBG section, which brings benefits to the proposed configuration. However, the size can be reduced if needed by truncating the left-hand side, as well as the top, of the PCB with no impact on performance. According to the figure of merit reported (FOM) in [18], which considers the achieved CP bandwidth, gain, polarization states, and number of antennas. The proposed antenna in this article offers a FOM of 50 compared to 9.75/5.1, 1.44, and 10 in [16,17,18], respectively, which confirms the potential and cost-effectiveness of the proposed antenna. It should be noted that the proposed configuration offers another distinct advantage of hybrid configurations since the bandwidth is also reconfigured from a wider single band in the CP modes to dual-narrower bands in the case of LP radiation.

## 8. Conclusions

In this paper, a cost-effective design of a mmWave antenna is proposed with hybrid reconfigurability, where planar feeding and biasing networks are employed to achieve a fully planar configuration. This was followed by measuring an antenna’s prototype with a close agreement between measurements and simulations. The proposed design focuses on achieving control over polarization, enabling seamless transitions between LHCP and RHCP senses as well as an LP mode by utilizing only two PIN diodes. This is combined with a reconfigurable bandwidth in which a single wider bandwidth is achieved in the CP states compared to dual-narrow bandwidths in the LP state. In addition, the presented planar structure features a biasing network consisting of coplanar strip lines that are integrated with photonic bandgap sections. This eliminates the need for traditional lumped elements, leading to enhanced radiation efficiency. Furthermore, the antenna’s design has been optimized by aligning the PIN diodes strategically, allowing for the utilization of a single CPS biasing circuit for the two PIN diodes. This contrasts with the conventional approach in the literature, which often necessitates two separate circuits for biasing two PIN diodes. This optimization simplifies the antenna design while improving its overall efficiency, resulting in an effective and streamlined configuration for a polarization and bandwidth reconfigurable mmWave loop antenna. The proposed antenna outperforms those reported mmWave polarization reconfigurable antennas in terms of the utilized number of PIN diodes, achieved impedance and CP bandwidths, and total efficiency. The proposed antenna represents a potential candidate in the design of multifunctional reconfigurable multiple input and multiple output, MIMO, antennas for 5G and B5G communications systems.

## Figures and Tables

**Table 1:** Dimensions of the proposed antenna.

Symbol	Parameter	Value (mm)
*R*_1_	outer loop radius	1.22
*R*_2_	parasitic loop radius	0.95
*t*_1_	outer loop width	0.28
*t*_2_	parasitic loop width	0.1
*Pad_l_*	pads length	8.7
*Pad_w_*	pads width	3.96
*l*_2_	transmission line width	0.25
*l*_3_	gap between the transmission line & pads	0.2
*a*_0_	antenna thickness	0.035
*h*	substrate thickness	0.508
*t*	reflector thickness	0.035
*Sub_x_*	substrate length	37
Δ*φ*_1_	outer loop’s gap 1	20°
Δ*φ*_2_	outer loop’s gap 2	20°

**Table 2:** RLC equivalent Circuit Parameters for PIN Diodes.

Parameter	Value
Total Capacitance C_T_	0.018 pF
Series Resistance R_S_	5.2 Ω
Parallel Resistance R_L_	15 KΩ
Total Inductive L_T_	1 nH
Forward Voltage	1.45 V

**Table 3:** Dimensions of the CPS bias line and PBG cells.

Parameter	Value (mm)	Parameter	Value (mm)
*A*	1.6	*W_b_*	1.48
*B*	3	*W_a_*	2.8
*C*	0.1	*pad_x_*	1.8
*D*	0.1	*pad_y_*	0.49
*E*	0.75		

**Table 4:** Reconfigurable loop antenna states for different polarization modes.

Case	1st PIN Diode	2nd PIN Diode	Current Direction	Polarization
*A*	ON	OFF	Clockwise	LHCP
*B*	OFF	ON	Counterclockwise	RHCP
*C*	OFF	OFF	---	LP

**Table 5:** Performance comparison between the proposed configuration and reported counterparts.

Ref.	Working States	Antenna	Elements Number	Switching Method	Frequency GHz	Gain dBci	S_11_ BW %	AR BW %	η_t %_	Size mm^2^
[12]	LHCP, LP	Patch	1	RF-MEMS	21	3.9	11.8	3	**	12 × 10
[13]	LHCP, RHCP, LP	Patch	1	Laser pulses	30	6.2	5.1,3.1	1.7	74/83	12.5 × 12.5
[14]	LHCP, RHCP, LP	Patch	1	4 PIN diodes	29	3,4	5.4	**	**	10.2 × 14.1
[15]	LHCP, RHCP	Patch	4	4 PIN diodes	28	6	11	4	51	31 × 34
[16]	LHCP, RHCP	T-Slot	10 × 1 2 × 2	Mechanical	29.5 30.5	16 13	3.3 10	3 3	80 80	97.3 × 18.1 26 × 26
[17]	LHCP, RHCP, LP	Butterfly	8 × 12	A ∠*φ* *	28	22	7	7	60	125 × 50
[18]	LHCP, RHCP	Patch	10	SPDT	30	13.9	17.7	8	75	22.5 × 61.75
This work	LHCP, RHCP, LP	Loop	1	2 PIN diodes	27/28	7.5	12.9	8	79	37 × 40

* Switching achieved by adjusting the magnitude and phase of the feeding single to each one of the 4 ports. ** Data not provided in the reference.

## Data Availability

Data are contained within the article.
